# Public and private sectors collective response to combat COVID-19 in Malaysia

**DOI:** 10.1186/s40545-021-00322-x

**Published:** 2021-05-03

**Authors:** Ching Siang Tan, Saim Lokman, Yao Rao, Szu Hua Kok, Long Chiau Ming

**Affiliations:** 1 School of Pharmacy, KPJ Healthcare University College, Nilai, Negeri Sembilan Malaysia; 2 Graduate School of Medicine, KPJ Healthcare University College, Nilai, Negeri Sembilan Malaysia; 3 Management School, University of Liverpool, Liverpool, UK; 4 Member of Synergy Integrity Consultancy and Management (SICM), Penang, Malaysia; 5 Pengiran Anak Puteri Rashidah Sa’adatul Bolkiah Institute of Health Sciences, Universiti Brunei Darussalam, Gadong, Brunei Darussalam

**Keywords:** Ministry of Health Malaysia, Private healthcare sectors, Insurance providers, COVID-19, Collaborative measures

## Abstract

Over the last year, the dangerous severe acute respiratory syndrome coronavirus 2 (SARS-CoV-2) has spread rapidly around the world. Malaysia has not been excluded from this COVID-19 pandemic. The resurgence of COVID-19 cases has overwhelmed the public healthcare system and overloaded the healthcare resources. Ministry of Health (MOH) Malaysia has adopted an Emergency Ordinance (EO) to instruct private hospitals to receive both COVID-19 and non-COVID-19 patients to reduce the strain on public facilities. The treatment of COVID-19 patients at private hospitals could help to boost the bed and critical care occupancy. However, with the absence of insurance coverage because COVID-19 is categorised as pandemic-related diseases, there are some challenges and opportunities posed by the treatment fees management. Another major issue in the collaboration between public and private hospitals is the willingness of private medical consultants to participate in the management of COVID-19 patients, because medical consultants in private hospitals in Malaysia are not hospital employees, but what are termed “private contractors” who provide patient care services to the hospitals. Other collaborative measures with private healthcare providers, e.g. tele-conferencing by private medical clinics to monitor COVID-19 patients and the rollout of national vaccination programme. The public and private healthcare partnership must be enhanced, and continue to find effective ways to collaborate further to combat the pandemic. The MOH, private healthcare sectors and insurance providers need to have a synergistic COVID-19 treatment plans to ensure public as well as insurance policy holders have equal opportunities for COVID-19 screening tests, vaccinations and treatment.

## Background

Over the last year, the dangerous severe acute respiratory syndrome coronavirus 2 (SARS-CoV-2) has spread rapidly around the world. Malaysia has not been excluded from this COVID-19 pandemic. This has had a detrimental effect on the economy and health system of the country, which has a population of 32 million people [1]. The newly reported COVID-19 daily cases skyrocketed from less than 100 cases in September 2020 to more than 5000 in January 2021 with a case fatality rate of 0.34% [1]. In the meantime, according to a report in the World Bank in December 2020, Malaysia’s economy is projected to contract by 5.8% in 2020 due to a sharp slowdown in economic activity caused by the COVID-19 and measures to contain its spread. Like many other countries, Malaysia is facing an unprecedented dual crisis. Currently, Malaysia is experiencing a second stringent nationwide lockdown (called a movement control order, MCO), following the first national lockdown in March 2020.

## Ministry of Health hospitals overwhelmed by the current resurgence of COVID-19 cases

An elevated number of vulnerable COVID-19 patients has put heavy pressure on public healthcare facilities in Malaysia. Currently the Malaysia healthcare service is delivered through two healthcare systems, which are the public and private systems. The public healthcare system is the main healthcare provider in the country, and the Ministry of Health (MOH) is the main national regulatory and policy-maker [2]. For financing, the public healthcare system is mainly supported by taxation and some other revenues. The private healthcare sector is underpinned by private healthcare insurance funds, patients’ out-of-pocket expenditures, and private and non-profit institutions [2].

A state of emergency was declared on 12 January 2021 by the Malaysian government to push for stricter control and drastic measures to better prepare for this critical nationwide disaster. Prior to the sudden spike of COVID-19 cases, all the COVID-19 patients were sent to public hospitals for treatment and isolation. However, the continuous increase in COVID-19 cases has overwhelmed the public healthcare system and overloaded the healthcare resources. In particular, ICU beds for COVID-19 patients at some major hospitals in the capital cities have reached maximum capacity.

## Synergy with private hospitals to treat COVID-19 patients

In terms of the cost of COVID-19 treatment to be borne by the patient, the fees at public facilities are currently affordable. The charges are similar to those for other non-communicable diseases as stipulated by the Federal Government Gazette: Order 2014—Medical Fees and Cost of Services [3]. However, due to the sudden increase in number of COVID-19 cases and almost full capacity of public hospitals, the government has had to reach out to the private sector to collaborate in the management of the pandemic.

Through the Prevention and Control of Infectious Diseases Act 1988, MOH Malaysia has adopted an Emergency Ordinance (EO) to instruct private hospitals to receive COVID-19 patients [4]. A fine of up to USD 1.23 million or jail time could be imposed if private hospitals refuse to receive category 1 and 2 COVID-19 patients under the EO [5]. At present, there are 210 private hospitals in Malaysia. The MOH has identified 130 private hospitals equipped with internal medicine specialties, and 95 hospitals are collaborating with the MOH to receive COVID-19 patients [6]. At a glance, a total of 1286 normal beds, 65 intensive care unit (ICU) beds and 54 ventilators could be leveraged throughout collaboration with private hospitals [6]. In line with the EO, the Malaysia government has allocated USD 24.7 million to private hospitals for the treatment of both COVID-19 and non-COVID-19 patients to reduce the strain on public facilities [7].

## The potential resources for management of COVID-19 and non-COVID-19 illnesses in private hospitals

There are huge resources in the private sector that can be potentially used in collaboration with the public sector. Taking into the consideration that the treatment cost at a private hospital is a fully out-of-pocket expense as well as the almost full capacity of public hospital beds, discussion has been initiated between the MOH and the Association of Private Hospitals of Malaysia (APHM) on the areas of collaboration and the policy of COVID-19 patients treated at private hospitals. During the pandemic, with the travel restrictions, the bed occupancy rate in private hospitals has been very low, and facilities underutilised. Private hospitals have been severely affected by the travel restrictions because they normally cater to medical tourists, including general treatment, surgeries, and oncology treatment. The treatment of COVID-19 patients at private hospitals could help to boost the bed and critical care occupancy. For example, KPJ Healthcare Bhd, one of the largest private hospital groups in Malaysia, immediately took heed of the government’s instruction and started receiving both COVID-19 and non-COVID-19 patients from public facilities in January 2021 [8]. Not only are the private hospitals treating COVID-19 patients, but non-COVID-19 patients can also be channelled to them, to ease the burden on public hospitals.

## Challenges and opportunities: collaboration between public and private hospitals

One of the major issues in the collaboration for the treatment of COVID-19 patients between public and private hospitals is the difference in the treatment fees. While the fees for public hospitals are very low as they are greatly subsidised by the government, private hospitals charge full fees, which can be as much as 10 times higher. Hence, APHM has urged insurance companies to revise their insurance policy to extend coverage of COVID-19 patients at private hospitals [9]. In response to this issue, the government should aid in reducing the operational cost from the private hospitals, in particular the expenses related to the management for COVID-19, such as testing, personal protective equipment and treatment, which have become the major hindrance to COVID-19 care in private hospitals. Similarly, government can set up a COVID-19 emergency fund, or work with insurance companies with the aim to relieve these financial burdens from the private hospitals.

One other major issue in the collaboration between public and private hospitals is the willingness of private medical consultants to participate in the management of COVID-19 patients, especially if the patients are from public hospitals. Medical consultants in private hospitals in Malaysia are not hospital employees, but what are termed “private contractors” who provide patient care services to the hospitals. Refusal of private medical consultants to participate is detrimental to the collaboration. Therefore, early engagement with the medical consultants is of paramount importance to increase their willingness to participate. To facilitate this, safety measures must be in place. In addition, equipment and drugs supply should be adequate, especially when the number of cases becomes overwhelming. This can be a key assurance for the healthcare professionals to work smoothly. On the other hand, difficult decisions have to be made about the allocation of the limited healthcare supplies between COVID-19-specific requirements and other health facilities, which includes, for example, not to support non-urgent treatments in the private hospitals. Finally, it is critical for medical professionals to understand that, without collective efforts between both public and private health sectors, the country’s public health and economy will be at fatal risk for an unforeseeable longer period.

## Other collaborative measures with private healthcare providers

The bed allocation for COVID-19 treatment at public and private facilities in Malaysia is 28,674 and 1286 beds, respectively [7]. This is a stark contrast compared to 45,478 COVID-19 patients currently under treatment (as of 29th January 2021). With a knee-jerk policy reaction, the Malaysian government has taken proactive measures to set up a warehouse or stadium as a COVID-19 Quarantine and Low-Risk Treatment Centre to cater for mild cases or asymptomatic COVID-19 patients [10]. The latest measure is to allow people with mild COVID-19 cases to self-isolate at home, especially if they have no symptoms [11]. The Malaysia Medical Association, which represents the medical fraternity, states that private medical clinics can help to monitor and care for COVID-19 patients by doing tele-conferencing and alleviate these patients’ fears.

Vaccination is one gigantic agenda in the pipeline. The first batch of vaccines was scheduled to arrive in Malaysia in February 2021 [12]. The Malaysian government provides free vaccination to the country’s citizens in stages. The effective rollout of a COVID-19 vaccination programme on a national scale calls for the involvement of community pharmacists to provide immunisation. In the last decade, community pharmacists in various countries have achieved much success in terms of safe and convenient vaccinations. Although a pharmacist-provided immunisation programme is not something new, this will be the maiden effort to have more than 2889 community pharmacies [13] in Malaysia administer the vaccine to the general public, and achieve the programme’s target of 75,000 daily injections [14]. Looking at the urgency of vaccinating older people in a timely manner and the need for a bolster dose within the recommended time frame, the existing 200-plus private hospitals should be roped in to expedite the vaccination rate.

## Conclusion

COVID-19 pandemic has revealed the existing and diverse nature of weaknesses of health systems across the world. Malaysia, like many other ASEAN countries, needs to strengthen its healthcare system, particularly to mitigate the COVID-19 cases in the country. Hence, the public and private healthcare partnership must be enhanced, and continue to find effective ways to collaborate further to combat the pandemic. The MOH, private healthcare sectors and insurance providers need to have a synergistic COVID-19 treatment plans to ensure public as well as insurance policy holders have equal opportunities for COVID-19 screening tests, vaccinations and treatment. Such plans would ensure smooth and efficient management of patients at both public and private hospitals. More importantly, the country will benefit from preparing such plan as a guide in event of a future disaster or pandemic.

As social distancing, mask-wearing, handwashing, and quarantining have all helped decrease the effects of the COVID-19 pandemic and will likely influence healthcare for the foreseeable future, the government should continue educating the public about COVID-19 to strengthen the public awareness about the precautionary measures and finally control the spread of COVID-19.

## Data Availability

Not applicable.

## Abbreviations

MOH: Ministry of Health
EO: Emergency ordinance
ICU: Intensive care unit (ICU)
APHM: Association of Private Hospitals of Malaysia
